# Abundant production of dimethylsulfoniopropionate as a cryoprotectant by freshwater phytoplanktonic dinoflagellates in ice-covered Lake Baikal

**DOI:** 10.1038/s42003-023-05573-9

**Published:** 2023-11-24

**Authors:** Kei Toda, Vladimir Obolkin, Shin-Ichi Ohira, Kentaro Saeki

**Affiliations:** 1 Department of Chemistry, Kumamoto University, 2-39-1, Kurokami, Kumamoto, 860-8555 Japan; 2 International Research Organization of Advanced Science and Technology (IROAST), Kumamoto University, 2-39-1, Kurokami, Kumamoto, 860-8555 Japan; 3 Laboratory of Hydrochemistry and Chemistry of the Atmosphere, Limnological Institute, Russian Academy of Sciences Siberian Branch, 3 Ulan-Batorskaya St., Irkutsk, 664033 Russia; 4 Department of Chemistry, Biology and Marine Science, University of the Ryukyus, 1, Senbaru Nishihara, Okinawa, 903-0213 Japan

**Keywords:** Freshwater ecology, Ecosystem ecology, Small molecules

## Abstract

Phytoplanktonic dinoflagellates form colonies between vertical ice crystals during the ice-melting season in Lake Baikal, but how the plankton survive the freezing conditions is not known. Here we show that the phytoplankton produces large amounts of dimethylsulfoniopropionate (DMSP), which is best-known as a marine compound. Lake-water DMSP concentrations in the spring season are comparable with those in the oceans, and colony water in ice exhibits extremely high concentrations. DMSP concentration of surface water correlates with plankton density and reaches a maximum in mid-April, with temperature-dependent fluctuations. DMSP is released from plankton cells into water in warm days. DMSP is a characteristic osmolyte of marine algae; our results demonstrate that freshwater plankton, *Gymnodinium baicalense*, has DMSP-producing ability, and efficiently uses the limited sulfur resource (only 1/500 of sea sulfate) to survive in freshwater ice. Plankton in Lake Baikal do not need an osmolyte, and our results clearly indicate that DMSP plays a cryoprotective role. DMSP, although a characteristic marine compound, could also be an important zwitterion for algae of other boreal lakes, alpine snow, and glaciers.

## Abstract (web summary)

DMSP potentially serves as a form of cryoprotection for freshwater dinoflagellates during the time of ice melting in Lake Baikal although sulfur content of the lake water is much lower than seawater and algae do not need an osmolyte in freshwater ice.

## Introduction

Dimethylsulfoniopropionate (DMSP) is a sulfur-containing zwitterion and representative osmolyte produced by marine phytoplankton. Dimethyl sulfide (DMS), formed by DMSP cleavage ^1–3^, is released into the atmosphere with diurnal flux variations^4^. The release of DMS into the atmosphere causes the characteristic smell of the ocean, plays an important role in the formation of cloud condensation nuclei, and contributes to the global biogeochemical sulfur cycle^5–8^. DMS and DMSP are also important signal molecules for predatory behaviors of marine copepods, fish^9,10^, and whales^11^. Marine algae produce DMSP for self-protection against the osmotic pressure of saline water^1,12,13^, using elemental sulfur from sulfate, which is abundant in seawater. As well as its role in osmotic pressure control, DMSP is considered to have multiple functions in marine algae^2^, such as an antioxidant^14^ and excess-energy acceptor^15^. The DMSP concentration in euphotic-zone seawater typically varies from 10 to 200 nM^16–19^. DMSP has also been detected in high-salinity lakes;^20,21^ however, it is generally not considered to be present in freshwater, with Lake Kinneret, located below sea level in Israel, being the only freshwater lake in which the existence of DMSP has been reported^22^. Ginzburg et al. showed that Lake Kinneret plankton produced DMSP abundantly during cultivation;^23^ thus, even freshwater plankton may have the ability to produce DMSP.

Phytoplankton in cold oceans produce greater amounts of DMSP than warm-ocean plankton. Several studies have reported DMSP in Antarctic^24–26^ and Arctic^27^ sea ice, and DMSP concentrations of 25–800 nM have been measured in Antarctic sea-ice cores^28^. The higher production of DMSP in cold oceans was explained in previous studies as cryoprotection of the plankton. The DMSP content of plankton increases with decreasing cultivation temperature^29–31^. Antarctic macroalgae also produce more DMSP at lower cultivation temperatures^32^. The higher osmolarity of the hypersaline environment of sea-ice brine channels may be another reason for the high DMSP production in this setting^33^. Wittek et al. cultivated Antarctic prymnesiophytes^34^ and sea-ice diatoms^35^ with covarying salinity and temperature (34‰ at 4 °C, 75‰ at –2.3 °C, and 100‰ at –3.9 °C). These results suggested a cryoprotectant role of DMSP in addition to an osmoprotectant role for prymnesiophytes, but a cryoprotectant role was not relevant for the sea-ice diatoms; thus, a cryoprotectant role of DMSP for plankton has not been clearly confirmed. A reason for this lack of confirmation is that DMSP simultaneously fulfils a number of functions. A demonstration that DMSP is abundantly produced at low temperature with few other factors would be strong evidence of a cryoprotectant role.

**Fig. 1:**
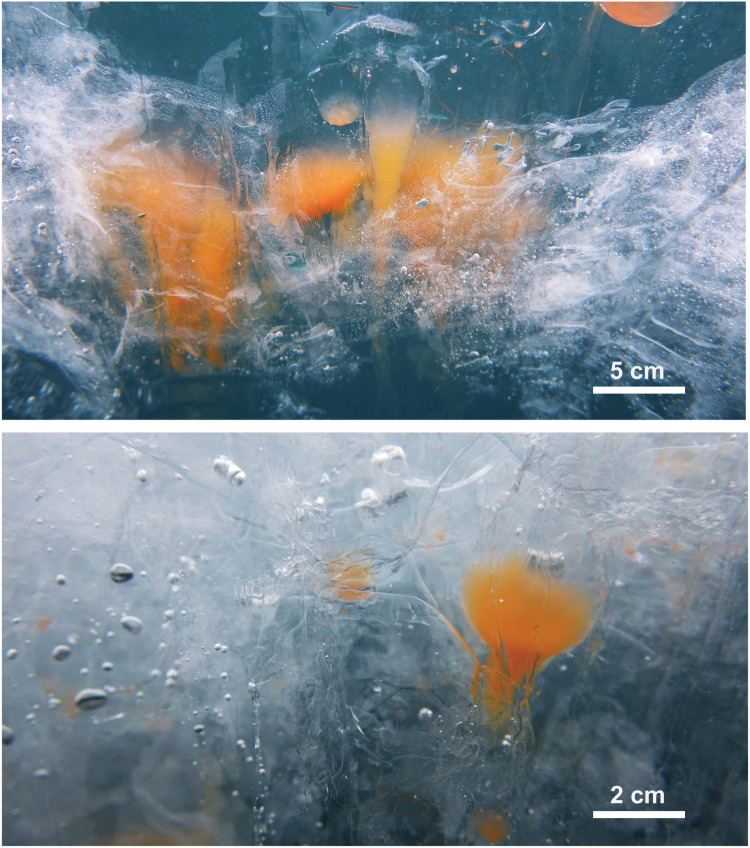
*Gymnodinium* colonies growing in ice. The orange masses are crowds of the planktonic dinoflagellate *Gymnodinium baicalense*. Individual plankton cells are approximately 30 μm in diameter. Plankton growing in cavities between vertical ice crystals increase during the day to form columnar colonies in ice. Ice holes occur at the top of the columnar water, through which plankton can move out to the bulk surface water.

We hypothesized that DMSP may be produced by freshwater algae for cryoprotection, despite the lower sulfur content and lack of a need for an osmolyte in freshwater. While surveying sulfur gas emissions from a pulp plant on the shore of Lake Baikal in Russia^36^, we heard from local people that DMS-like odors can be detected from the lake water in the ice-melting season. Lake Baikal is the clearest freshwater lake in the world^37^, and the formation of DMS from pollution is therefore unlikely. DMSP might be naturally produced in the Baikal water and converted to DMS. Lake Baikal, the oldest and largest (by volume) lake in the world, has two plankton bloom seasons: one in August and the other between March and April. Diatom species such as *Aulacoseira baicalensis* have been observed in March on the ice-bottom surface^38^ and in ice cores^39^. Dinoflagellates such as *Gymnodinium baicalense* bloom mainly in April^39,40^. Annenkova et al. genetically identified the plankton^41,42^ and illustrated an ice bottom covered with algae^43^. *Gymnodinium* colonies have been observed on ice-crack walls, as shown in Fig. 1. Ice cracks, formed by repeated thermal expansion during the day and contraction during the night, are good plankton incubators because the walls form a water–ice interface on which algae can settle; in addition, the walls receive more sunlight than the bottom of the thick ice. Marine dinoflagellates, including *Gymnodinium*, are known to be DMSP producers^44,45^, and the freshwater species *Gymnodinium baicalense* may produce DMSP during the spring blooming season.

We investigated the presence of DMSP in freshwater, starting our preliminary survey^46^ in 2012 in Lake Baikal, and performing detailed analyses in 2019. To evaluate DMSP production, ice holes were prepared in March, daily water samples were taken, and DMSP concentrations were measured onsite within an hour of sampling. The sulfate ion concentration of Lake Baikal freshwater is only 1/500 that of seawater, and sulfate is a source of elemental sulfur necessary for DMSP production. Furthermore, osmotic pressure control is not necessary for Baikal plankton. Detection of DMSP in Baikal water would be strong evidence for a cryoprotective role of DMSP. In this study, our aims were to determine whether high DMSP production occurs in Lake Baikal, even under such unfavorable conditions, and to evaluate daily variations in DMSP concentration during the plankton bloom. We analyzed both water from the holes (surface water) and water within the ice colony (colony water) to elucidate the mechanisms preventing water in the ice cavities from freezing and allowing the algae to bloom. This paper reports significant production of marine compound, DMSP, in the ice-covered freshwater lake.

## Results and discussion

### DMSP detected in Lake Baikal water

**Fig. 2:**
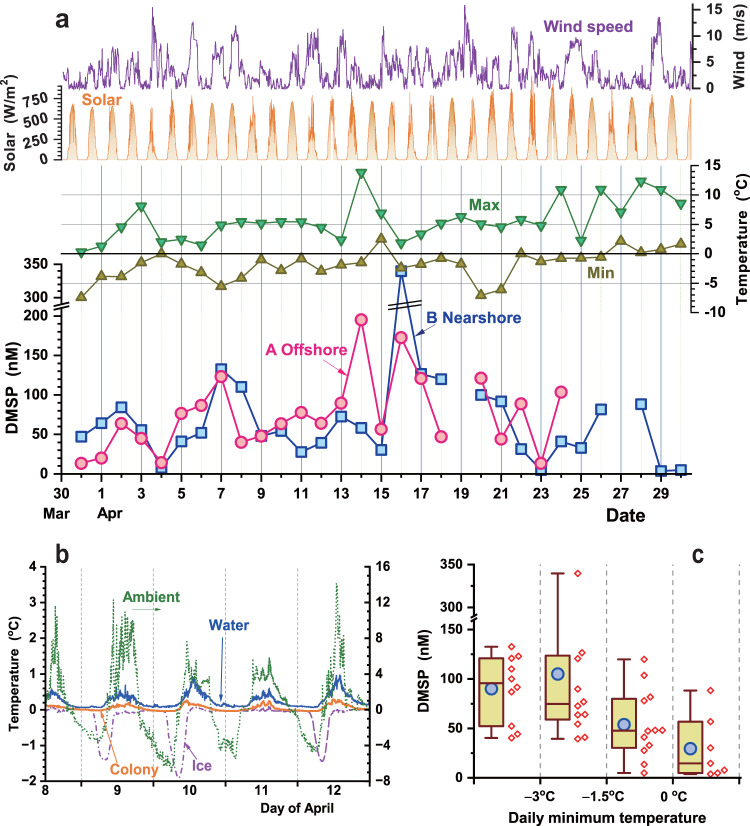
Daily fluctuations in DMSP concentration. **a** DMSP concentrations at offshore (point A, red circles) and nearshore (point B, blue squares) sites, and meteorological data. **b** Changes in ambient (green dot line), ice (violet chain line), surface water (blue solid line), and plankton colony (orange solid line) temperatures measured at point A. **c** Ranges of DMSP concentration at daily minimum temperatures below −3 °C (*n* = 10 independent surface water samples), between −3 and −1.5 °C (*n* = 12), between −1.5 °C and 0 °C (*n* = 12), and above 0 °C (*n* = 7). Boxes show interquartile ranges (25–75%). Blue circles and horizontal bars in boxes are average and median values, respectively. Bars above and below boxes are maximum and minimum values, respectively.

DMSP was detected in all samples obtained between the end of March and the beginning of May. Synchronized changes in DMSP concentrations were observed for the two sampling points, A offshore and B nearshore (Fig. 2a), even though they were 600 m apart and their plankton colonies were not connected. Our first measurements on day 1 already indicated the presence of DMSP, with a concentration at point B of approximately 50 nM. The DMSP concentrations at both sampling points exhibited daily fluctuations and maximum concentrations were reached in mid-April, with the highest DMSP value at point A being 195 nM on April 14, and that at point B being 340 nM on April 16, after which the DMSP concentrations gradually decreased. Offshore observations were discontinued on April 25 because reaching point A became dangerous as a result of melting ice and crack formation. Nearshore observations at point B were continued until the end of April, with DMSP concentrations of less than 5 nM recorded on April 29 and 30. In mid-May, water sampled from the shore contained only sub-nM DMSP concentrations. DMSP was therefore present in Lake Baikal for approximately 4 weeks, at a maximal concentration in mid-April.

The variations in ambient temperature 20 cm above the ice suface, inside the ice, in the surface water of the hole at 40 cm depth, and in the ice-cavity colony water, measured for 5 days at point A are shown in Fig. 2b. The minimum and maximum ambient temperatures during the 5 days were –6.9 and +14.2 °C, respectively, with diurnal changes ranging between 10.2 and 17.7 °C. In contrast, the other temperatures did not fluctuate much. The ice temperature was below zero (minimum –1.3 to –1.8 °C) in the morning and surface water temperatures rose to a maximum of ca. 1 °C in the afternoon. The colony water temperature was between the surface water and ice temperatures and remained at 0 °C during both day and night. The water in the vertical ice cavities is therefore likely to provide a stable and favorable environment for cryophilic algae to form colony as shown in Fig. 1. Under such constant temperature conditions, solar radiation accelerates photosynthesis and algae growth, and the absorbed heat augments ice melting, thereby maintaining or progressively expanding the colony water volume.

Box plots showing the distribution of DMSP concentrations at points A and B against four ranges of daily minimum temperature are provided in Fig. 2c. DMSP concentrations were high as 90 ± 34 and 105 ± 83 nM at minimum temperatures below –3 °C and between –3 and –1.5 °C, respectively, and decreased with temperature to 54 ± 35 nM between –1.5 and 0 °C and to 30 ± 32 nM above 0 °C. Median values decreased as 96, 75, 48, and 15 nM with increase in the minimum temperature range. The surface-water DMSP concentration was higher at lower morning temperatures.

### DMSP concentration and plankton

**Fig. 3:**
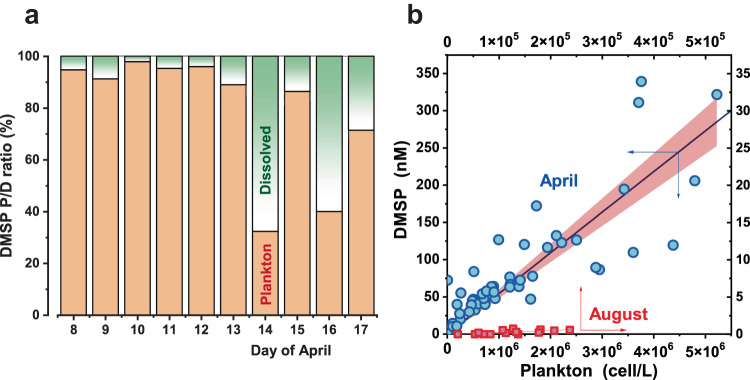
DMSP and plankton. **a** Ratio of DMSP in plankton to that dissolved in water. **b** Correlation between DMSP concentration and *Gymnodinium* density in April (blue circles) and August (red squares) (independent surface water samples *n* = 55 and 15, respectively). Note that the plot ranges of the *x-*axis and *y*-axis for August are 1/10 of those for April, but the *x*/*y* ratio remains the same.

The concentration ratios of DMSP in plankton and DMSP dissolved in water are plotted in Fig. 3a. A dramatic change in the DMSP ratio was observed on April 14, which was a warm day with a maximum ambient temperature of 13.5 °C. Although DMSP was mostly contained in plankton until April 13, two-thirds of the DMSP was dissolved in the water on April 14 (Fig. 3a). This high ratio of dissolved DMSP was subsequently maintained. Although dissolved DMSP might have been overestimated as a result of release from the cells during filtration^31,47^, the ratio of dissolved DMSP obviously increased on the warm day, on which the surface water temperature must have been above freezing. Following the temperature increase, DMSP became redundant and might have been exudated from the cells. Ice melting on warm days resulted in release of interstitial plankton from 0 °C colony water into higher-temperature surface water. This plankton release may have accelerated the exudation. A similar process, an increase in the ratio of dissolved DMSP in Arctic sea-ice algae during melting, has been explained by salinity changes caused by plankton release from higher-salinity porewater into seawater^31,48^. However, the ion concentrations of colony/surface waters of Baikal were very low compared to seawater, as discussed below, and possible active exudation can be thought to have resulted from a temperature change rather than a pressure change. Cell lysis is another major process that could produce dissolved DMSP, and might have been caused by the temperature increase. April 14 was a warm day and the time of the maximum DMSP concentration, which was followed by a decline. Algae experiencing overcrowding might have released DMSP into the water as a result of cell death under nutrient-limited or stressful conditions^49^ on those days.

The DMSP concentration briefly decreased on April 15 (from 195 to 57 nM and back to 172 nM the next day at point A; Fig. 2a), when the ambient temperature did not decrease into the freezing range (minimum +3.0 °C). Drops in DMSP concentration were observed also on April 4 during bloom growth and on April 22 and 23 during the decay period; the ambient temperature was above the freezing point in the morning on those days. Thus, the plankton abundance and DMSP concentration of surface water is high at lower temperatures. Whether the ambient minimum temperature is below or above the freezing point is an indicator of the plankton and DMSP trends of the surface water.

DMSP concentrations in surface water are plotted against plankton densities (cell L^–1^) of *Gymnodinium* in Fig. 3b, including data obtained in August. The DMSP concentrations of lake surface water correlated well with plankton density of *Gymnodinium* (Eqs. 1 and 2). The DMSP concentration increased with increasing plankton abundance, with a correlation coefficient (*R*) of 0.850.

1 $$\begin{array}{ccc}{{{{{\rm{April}}}}}}: & y=(54.6\pm 3.1)\times {10}^{{{{{{\rm{\hbox{-}}}}}}}6}x & R=0.850,n=55,p \, < \, 0.0001\end{array}$$

2 $$\begin{array}{ccc}{{{{{\rm{August}}}}}}: & y=(2.35\pm 0.40)\times {10}^{\mbox{-}6}x & R = 0.662, n=15, p=0.00693\end{array}$$

In April, *Gymnodinium* was the major plankton taxon, with *Rhodomonas sp*. (Cryptophyceae), *Cryptomonas* (Cryptophyceae), *Ulnaria radians* (diatom), and *Nitzschia graciliformis* (diatom) observed as minor species. April bloom water contained abundant plankton, and the *Gymnodinium* density in summer was only 1/20 that of the spring bloom. The slope of the fitted line for the April data is 23.2 times greater than that for August, with DMSP per plankton values of 54.6 ± 3.1 and 2.35 ± 0.40 fmol cell^–1^ for April and August, respectively (Fig. 3b). DMSP cell concentrations in incubated marine plankton cultures of 0.0075–6.56 fmol cell^–1^ for diatoms and 25.2, 109, and 304 fmol cell^–1^ for dinoflagellates^50^ have been reported, indicating that dinoflagellates produce more DMSP than diatoms^51,52^. Caruana and Malin constructed a database of DMSP of marine dinoflagellates, and found a range of DMSP content spanning five orders of magnitude, from 0.1 to 14700 fmol cell^–1^ with a median value of 170 fmol cell^–1^ ^53^. Note that some data are of total DMSP concentration, because DMSP is mostly present in particles, and the concentration of DMSP in particles can be approximated to the total DMSP concentration. The DMSP concentration of *Peridinium gatunense*, a dinoflagellate of Lake Kinneret, increased during cultivation and was ca. 5 fmol cell^–1^ or less at 40–70 days and ca. 40 fmol cell^–1^ at 90 days, at the end of growth, whereas DMSP concentrations of Lake Kinneret water were very low^23^. *Gymnodinium baicalense* produced DMSP at the level of marine dinoflagellates in the natural freshwater environment of Baikal in April.

These results suggest that dinoflagellates have the genetic ability to produce DMSP and synthesize the chemical when they are exposed to stresses, which may be osmotic pressure stress, oxidative stress, and/or freezing stress. DMSP production is known from dinoflagellates, including marine *Gymnodinium* species. The pathway to produce DMSP is likely an ancestral feature of the genus, so they have re-purposed an ancestral pathway rather than acquired the pathway by some other means. Lake Kinneret *Gymnodinium* produce DMSP in cultivation, and Baikal *Gymnodinium* produce DMSP in the ice-covered lake.

### Chemical composition of colony water in ice

Columnar cavities between vertical ice crystals serve as a natural growth medium for plankton incubation (Fig. 1). Comparison of the chemical composition of colony water and hole surface water (Table 1) indicated that colony water contains higher levels of sulfur zwitterions, including methionine and cysteine as well as DMSP. Cysteine was not detected in surface water, whereas colony water exhibited cysteine concentrations of 12 ± 9 nM. Methionine concentrations were higher, 431 ± 327 nM in colony water and 10 ± 7 nM even in surface water. Methionine is an important precursor for DMSP synthesis and for protein production in marine algae^2,45,54^. The high content of methionine in colony water is consistent with the DMSP synthesis mechanism. Of the sulfur zwitterions, DMSP displayed the highest concentrations of 636 ± 326 nM in colony water and 41 ± 36 nM in surface water. One colony sample exhibited a DMSP concentration exceeding 1000 nM. Dimethyl sulfoxide (DMSO) was also detected, with a concentration in colony water three times greater than that in surface water, suggesting that cleavage of DMSP to DMS, and transformation of DMS to DMSO, occurs even in the colony water. DMSO formation has previously been investigated only for ocean^55–57^ and estuary waters;^58^ our results demonstrate DMSO production in freshwater. DMSO also acts as an osmolyte, cryoprotectant^59^, and antioxidant^14^. The dimethyl sulfur chemical system (DMSP/DMS/DMSO) is an effective cryoprotection and antioxidation system for marine algae, and may provide the same functions in freshwater plankton blooms.

**Table 1:** Contents of sulfur compounds and inorganic ions of colony and ice hole surface waters.

	Colony water (*n* = 7)	Surface water	
		Ice hole (*n* = 24)	Literature
Sulfur compounds (nM)			
DMSP	636 ± 326	40 ± 36	
Methionine	431 ± 327	10 ± 7	
Cysteine	12 ± 9	0.2	
DMSO	23 ± 34	8.6 ± 9.4	
Inorganic ions (μM)			
Cl^–^	38 ± 8	29 ± 12	11
NO_3_^–^	6.7 ± 1.8	2.6 ± 3.0	6.4
SO_4_^2–^	38 ± 7	52 ± 23	54
NH_4_^+^	116 ± 64	44 ± 104	1
Na^+^	344 ± 68	130 ± 38	143
K^+^	130 ± 15	217 ± 275	23
Mg^2+^	598 ± 156	145 ± 54	123
Ca^2+^	264 ± 54	439 ± 107	407

Values indicating in the right end represent typical ion concentrations of Lake Baikal freshwater from the literature^46^. Colony > surface: Mg^2+^, NH_4_^+^, NO_3_^−^, Cl^−^, DMSO, cysteine, methionine, and DMSP. Surface > colony: Ca^2+^, K^+^, and SO_4_^2−^.

**Fig. 4:**
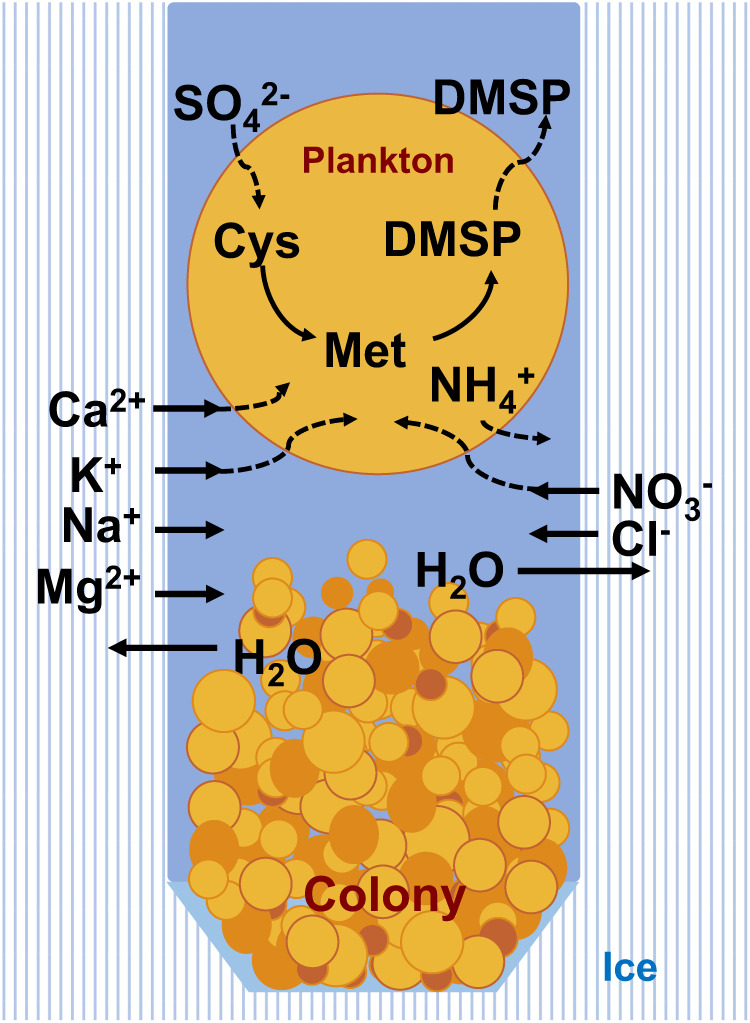
Flow of chemicals in the ice–water–plankton system. Cys: cysteine, Met: methionine. Colony > surface: Mg^2+^, NH_4_^+^, NO_3_^−^, Cl^−^ ions are concentrated by thawing and refreezing. Ca^2+^, K^+^, and SO_4_^2−^ ions are consumed to be lower. Sulfur element of sulfate is used to produce cysteine, methionine, DMSP and DMSO.

Evaluation of inorganic ion concentrations revealed higher inorganic ion concentrations in colony water than in surface water, except for potassium, calcium, and sulfate ions. Repeated thawing and re-freezing within the vertical ice cavities leads to ion enrichment^60^ as ice preferentially melts from mineral-rich parts, and ions remain in the water during re-freezing (Fig. 4). The lower potassium and calcium ion levels may be attributed to the consumption of these ions by plankton^61^. Ammonium ion levels in both the surface and the colony water were much higher than the normal level for Lake Baikal as a result of metabolism of plankton, and oxidation of the abundant ammonium resulted in high nitrate concentrations in the colony. Although the sulfate concentration in surface water (52 ± 23 μM) was the same as the reported Baikal concentrations^46^, the sulfate concentration in colony water (38 ± 7 μM) was approximately 30% lower than that of the surface water, suggesting that sulfate in colony water is consumed to produce sulfur-containing amino acids, proteins, and DMSP.

Sunlight absorption by plankton increases water temperature, which accelerates daytime ice-melting. The ion-rich water in the ice cavities is a source of minerals for plankton. This ion-rich water tends not to freeze, and DMSP protects plankton from freezing at night. The colony water temperature is almost constant at 0 °C (Fig. 2b), even when the ambient and ice temperatures vary from −5 to 10 °C, thus allowing plankton to bloom in the ice.

Inorganic ions in colony water were present in concentrations 1.5 times those of ions in surface water (Table 1). Kameyama et al. proposed that higher osmolarity of sea-ice brine channels may be another reason for the high DMSP production;^33^ however, the salt concentration in Baikal ice channels was still 1/400 that of seawater, and the resulting osmotic pressure was considered to be negligible. In addition to cryoprotection, another role of DMSP, antioxidation, may be important for the Baikal plankton. Cells are densely packed in small cavities, resulting in stressful conditions during the blooms developed in the ice channels (Fig. 1). Data were limited, but the DMSP/plankton ratios of colony waters at the beginning of blooming (63, 33, and 24 fmol/cell) were at the same approximate level as in the surface water (55 ± 3 fmol/cell), and increased to higher levels in cell water (57, 114, 251, and 177 fmol/cell) in the middle of April. This phenomenon is similar to that observed in cultivated Lake Kinneret plankton, as stated in the previous subsection. Overcrowding stress might accelerate the DMSP production.

### Comparison of DMSP concentrations and DMSP/sulfate ratios among oceans, hypersaline lakes, and Lake Baikal freshwater

We compared the Lake Baikal freshwater DMSP concentrations obtained in this study with reported DMSP concentrations in seawater (Table 2). The average surface-water DMSP concentration for the two Baikal sampling points was 71 ± 56 nM, which is the same order of magnitude or higher than the typical DMSP concentrations in low- and middle-latitude oceans. Atlantic Ocean DMSP concentrations of approximately 18 nM have been reported in most of the ocean (between latitudes 30°S and 40°N), and higher concentrations have been observed in high latitudes of both hemispheres (maxima of 90 nM around 40°S and 70 nM around 50°N)^62^. Higher DMSP concentrations have been detected in ice-covered seas of the polar regions (26–253 nM)^27,28,63^. The DMSP concentrations of Baikal surface water in the spring bloom season were comparable to those of ice-covered seas. DMSP is produced not only in seawater but also in hypersaline lake water, with the waters of Lakes Humboldt, Patience, and Waldsea containing sulfate ion concentrations of 19–172 mM and DMSP average measured concentrations of 4–38 nM. The sulfate concentrations of Lakes Big Quill, Little Manitou, and Chaplin are one order of magnitude higher than that of seawater, with DMSP concentrations of 43–800 nM. The higher the salinity of the saline lake water and seawater, the higher the DMSP level, which is consistent with the results of cultured algae experiments with artificial salinity variations^2^. DMSP is not known as a freshwater compound, but DMSP has been detected in Lake Kinneret surface water at concentrations of several nanomolars^22^, with a sulfate concentration one order of magnitude higher than that of Lake Baikal. Although DMSP concentrations are correlated with salt and sulfate concentrations, high concentrations of DMSP were observed in Lake Baikal in spring, despite the low sulfate concentration. The sulfate concentration in Lake Baikal water was only 0.052 mM, three and four orders of magnitude lower than that of seawater and hypersaline lake water, respectively. Despite the limited sulfur availability, DMSP is efficiently produced in Lake Baikal. The DMSP concentrations, sulfate concentrations, and DMSP/sulfate ratios are provided in Table 2. DMSP/sulfate ratios are <0.001‰ in oceans and super-saline lakes, and <0.01‰ in sea ice. In contrast, DMSP/sulfate ratios in Lake Baikal surface water in April were 1.37 ± 1.08‰, three orders of magnitude greater than those of seawater. The algae colony water DMSP/sulfate ratio was a further order of magnitude higher, at 16.7 ± 8.6‰. Thus, *Gymnodinium baicalense* efficiently takes up sulfate from water to produce DMSP. Although carbon stored as carbohydrate in algal cells is consumed during synthesis of amino acids and protein, sulfur is obtained from water sulfate when required, as has been experimentally confirmed for both marine and freshwater plankton^64^. The observation of DMSP production by cultivated Lake Kinneret plankton^23^ is also evidence showing that freshwater plankton have the ability to take sulfur from water to produce DMSP. This ability to absorb sulfate allows plankton to survive in the ice-water environment of Baikal. Although the sulfate reduction required to produce sulfur-containing organic compounds is an energy-consuming process^2^, the plankton effectively synthesize methionine and DMSP by using sulfur obtained from the limited supply of sulfate ions.

**Table 2:** DMSP/sulfate ratios of seawater, sea-ice cores, super-saline lake water, and fresh lake water.

Place	Sulfate (mM)	DMSP (nM)	DMSP/sulfate Ratio (‰)	Note
Sea				
Atlantic (S30–N40°)	(28)^a^	17	0.00061	Aumont et al. 2002^62^
(S40°)		92	0.0033	
(N50°)		70	0.0025	
Ariake Sea		18	0.00066	Nagahata et al. 2013^68^
Amakusa		15	0.00052	Iyadomi et al. 2016^4^
Yellow Sea		25	0.00090	Yang, et al., 2012^69^
South China Sea		10	0.00036	Yang, et al. 2008^70^
Ice-covered sea				
Arctic surface water		70	0.0025	Galí and Simó 2010^63^
Arctic under ice		26	0.00093	Galindo et al. 2016^27^
Antarctic				
0.5 -0.7 m thick ice 0.7-1.2 m thick ice		253 173	0.0090 0.0062	Trevena and Jones 2006^28^
Saline lake				
Humboldt	19	4	0.00021	Canadian saline lakes Richard et al. 1994^20^
Patience	48	10	0.00021	
Waldsea	172	38	0.00022	
Big Quill	589	800	0.0014	
Little Manitou	589	43	0.00007	
Chaplin, east	927	110	0.00012	
Chaplin, west	802	340	0.00042	
Ice-covered saline lake				
Bonney, west, 10-17 m	NA	15		Lee et al. 2004^21^
Bonney, east, 10-18 m	NA	3		
Freshwater lake				
Kinneret, surface	0.54	5.7	0.011	Sela-Adler et al. 2016^22^
Baikal, surface	0.054	0.26	0.0048	This work, in summer
Ice-covered freshwater lake				
Baikal, surface	0.052	71	1.4	This work
Baikal, colony	0.038	636	17	

The listed sulfate and DMSP concentrations are average values of data from the literature or our results.

^a^The seawater sulfate concentration (28 mM) is the typical concentration as given in the literature^71^.

## Conclusions

In this paper, we demonstrate abundant production of DMSP production in Lake Baikal in the ice-melting season where *Gymnodinium* breeds in channels between ice crystals and surface water. The DMSP concentration is higher on cold days and is particularly low when the minimum temperature is above the freezing point. DMSP acts as an antioxidant as well as an osmoregulator and cryoprotectant. As osmotic pressure stress is negligible in Baikal freshwater, the present results are evidence demonstrating that DMSP is produced for cryoprotection. These findings are expected to be validated in future by culture experiments at different temperatures and by identifying the genes responsible for DMSP production in freshwater plankton. As well as increased DMSP production, increased expression of these genes at lower temperatures may occur. Furthermore, DMSP might be produced also by algae of other boreal lakes, alpine snow, and glaciers, as a common zwitterion for freshwater ice blooming.

## Methods

### Water sampling and sampling strategy

DMSP sampling and analyses were performed in the southwest of Lake Baikal near the mouth of the Angara River from March 29 to May 13, 2019. The main sampling points were holes prepared on the ice. The initial ice thickness (at the end of March) was ca. 70 cm. Details of meteorological data are included in Fig. 2 of the paper. The ambient temperature was below freezing point every morning until April 20, after which daily minimum temperatures were mostly above freezing point.

Two holes, approximately 0.7 m in diameter, were drilled approximately 1 km east of the Baikal Museum for fixed-point observations. Offshore point A was drilled 700 m from shore at N 51°51’29.8”, E 104°50’04.2”, and nearshore point B was drilled 70 m from the shore at N 51°51’52.1”, E 104°50’27.8”. Water depths were 650 m at point A and 10 m at point B.

Surface water was sampled from the holes using plastic bottles at 3–5 cm depth to avoid collection of floating substances. The dominant plankton species in water samples was the dinoflagellate *Gymnodinium baicalense*, which was present at abundances of 20–4800 cell/mL in the surface water of the holes. The bottles were capped under water to eliminate headspace and were transported to the Baikal Museum in a light-shielded bag. The last sample on the ice was obtained on April 30, and shore water analysis continued until May 13. Fixed-point measurements were repeated almost every day, and surface-water samples were obtained on 24 days from point A (offshore) and on 29 days from point B (nearshore). Measurements at Point A ceased on April 24 because the site was no longer accessible. DMSP measurements were conducted twice for each sample. DMSP concentration changes from the start to the end of a plankton bloom were obtained. The correlation of DMSP concentration with plankton density was analyzed on the basis of 55 measurement data. For comparison, a summer campaign was carried out near point A during August 5–19 to measure DMSP concentration and plankton counts in 15 surface-water samples taken from a boat.

Colony water was sampled by inserting a glass pipet into a columnar water channel formed on an ice wall and transferred in a 15-mL plastic tube. In the pipetting process, surface water came in the colony to dilute the colony water somewhat. The sampled colony waters contained *Gymnodinium* at 21000–93000 cell/mL.

The main purpose of this study was to investigate whether plankton in fresh water produce DMSP during the ice season. The first stage of the work was to determine whether DMSP could be detected in the lake water. As the second stage, instead of sample-size calculation, a series of DMSP concentration trends was obtained from the beginning to the end of the bloom. Monitoring was performed at two locations simultaneously to provide better data reliability. The same trends were observed at both locations, and the sampling points were sufficiently far apart to avoid bloom interaction. The correlation between DMSP concentration and plankton density was obtained for sample waters taken from the two fixed sampling points, other holes, and clacks. The measurements were performed for more than 50 samples to obtain the correlation in the blooming season.

### On-site measurement of DMSP

The necessary equipment and measurement instruments were set up in March 2019, and measurements commenced on March 31. Sample water was treated for DMSP analysis within an hour of sampling in the Baikal Museum. Sample bottles were flipped a few times immediately before measurement. A 3–12 mL sample was taken in a bubbling tube and diluted to 12 mL. Purified air was introduced to eliminate dissolved DMS. Colony water samples were measured in the same way but after appropriate dilution. Then, 2 mL of 5 M NaOH solution was added to the sample with a syringe needle inserted through the bubbler plug^4^. After 60 min hydrolysis, DMS, produced from DMSP, was vaporized by air bubbling and introduced to a DMS analyzer, a single-column trapping/separation–chemiluminescence detection system. Details of the system are provided in the references^36,65^. Briefly, after passing through a Nafion dryer to dehumidify the bubbling air, vaporized DMS was trapped in a ceramic tube column packed with silica gel (Davison Grade 12, 60–80 mesh, Supelco, Merck, Darmstadt, Germany). The tube was stepwise heated with flowing nitrogen carrier gas to allow separation from other volatile sulfur compounds. The desorbed DMS gas subsequently reacted with ozone to produce chemiluminescence^66,67^. Data from the dimethyl sulfide (DMS) analyzer were collected by using a data logger (USB-1408FS, Measurement Computing).

DMSP concentrations were obtained without filtering, unless otherwise specified, and correspond to total DMSP concentrations, i.e. the sum of DMSP dissolved in water and in plankton. For Fig. 3a data, each water sample was gently filtered through a glass-fiber filter (Whatman GF/A ф47 mm). The filtrate was treated as descibed above to obtain the dissolved DMSP concentration.

For plankton density analysis, water samples were fixed with Utermohl’s solution, well-vortexed, and pipetted into 0.1-mL counting plates (MPC-200, Matsunami Glass, Kishiwada, Japan). *Gymnodinium* cells were counted under a microscope. Three measurements were averaged for each sample.

### Laboratory analysis of inorganic ions and sulfur-containing amino acids

For the chromatography measurements, the water samples were centrifuged, and the supernatants were filtered before analysis to remove particles. Then, water samples were analyzed using ion chromatographs, 761 Compact IC (Metrohm, Herisau, Switzerland) for anions and Dionex ICS-1100 (Thermo Fisher Scientific, Sunnyvale, CA) for cations. DMSO, methionine, and cysteine were analyzed using a liquid chromatograph-electrospray ionizer-tandem mass spectrometer (HPLC-MS 8400, Shimadzu, Kyoto, Japan) equipped with a hydrophilic interaction liquid chromatography column (VC-50 2D, Shodex, Tokyo, Japan). The eluent consisted of a 50-mM formic acid and acetonitrile (9:1) mixture in isocratic mode at a flow rate of 0.2/mL. DMSO, cysteine, and methionine were eluted at retention times of 2.3, 2.8, and 3.5 min, respectively, and detected in the selected reaction monitoring mode by monitoring precursor ions at 79.15 *m*/*z* and product ions at 64.15 *m*/*z* for DMSO, at 122.0 *m*/*z* and 76.1 *m*/*z* for cysteine, and at 150.0 *m*/*z* and 133.0 *m*/*z* for methionine.

### Meteorological data

Meteorological data were kindly provided by the hydrological laboratory of the Limnological Institute, Siberian Branch, Russian Academy of Science. The data were measured at the Listvyanka harbor, which is located 2 km southeast of the observation points. Temperatures at point A were monitored from April 8 to 13 using temperature loggers (TM-9017SD, Fuso, Tokyo). The temperature probes (platinum 100 Ω) were inserted in the ice-wall colony, in water at a depth of 40 cm, and in the ice at a depth of 10 cm. The ambient temperature was monitored with a probe placed in the shade 20 cm above the ice surface. The temperatures were logged every 5 min.

### Statistics and Reproducibility

The study was continued for a month to obtain day-to-day variations of DMSP concentration. We performed several preliminary surveys from 2011 onward, from which we came to know that DMSP levels are low in March and high in mid-April. Thus, the measurement period was set as the end of March until the ice melted to cover the entire blooming period, and we observed the variations as expected. Sampling was performed at a fixed time, 10–11 am, to minimize diurnal variation effects. To confirm the tendency, two sampling points were used; the same trends were obtained from the two independent points. DMSP and plankton were measured twice and thrice, respectively, and the obtained values did not exhibit unreasonable differences.

DMSP concentration was plotted against daily minimum temperature. Forty-one data for water samples obtained from points A and B were divided into four groups according to the minimum temperature ranges (*T* < − 3 °C, −3 °C < *T* < − 1.5 °C, −1.5 °C < *T* < 0 °C, and 0 °C < *T)*, and the mean, lower quartile, median, upper quartile, and lowest and highest values were obtained for each temperature range by using Origin software.

Gymnodinium was counted in triplicate for 55 samples in April and DMSP concentration was plotted against plankton density. For comparison, 15 sample data of August were analyzed in the same way. Regression curves were obtained for the two-season samples with the intercept set at zero. Regression equations and 95% confidence intervals were obtained by Origin software.

### Reporting summary

Further information on research design is available in the Nature Portfolio Reporting Summary linked to this article.

### Supplementary information

Supplementary Data 1

Reporting Summary

Description of Supplementary Materials

## Data Availability

The authors declare that the data supporting the findings of this study are available within the paper. The numerical source data underlying all figures are available from Supplementary Data of this paper. https://www.nature.com/articles/s42003-023-05573-9.
