# Supplementary material for: Abundant production of dimethylsulfoniopropionate as a cryoprotectant by freshwater phytoplanktonic dinoflagellates in ice-covered Lake Baikal
Source: Commun Biol. 2023 Nov 24;6:1194. doi: 10.1038/s42003-023-05573-9 (PMC10674015; doi:10.1038/s42003-023-05573-9)
Supplement: Supplementary file 2 — Reporting Summary [file 42003_2023_5573_MOESM2_ESM.pdf]

nature portfolio

Corresponding author(s): Kei Toda

Last updated by author(s): Oct 10, 2023

Reporting Summary

Nature Portfolio wishes to improve the reproducibility of the work that we publish. This form provides structure for consistency and transparency in reporting. For further information on Nature Portfolio policies, see our Editorial Policies and the Editorial Policy Checklist.

Statistics

For all statistical analyses, confirm that the following items are present in the figure legend, table legend, main text, or Methods section.

n/a	Confirmed	
☐	☒	The exact sample size (n) for each experimental group/condition, given as a discrete number and unit of measurement
☐	☒	A statement on whether measurements were taken from distinct samples or whether the same sample was measured repeatedly
☐	☒	The statistical test(s) used AND whether they are one- or two-sided<br>*Only common tests should be described solely by name; describe more complex techniques in the Methods section.*
☐	☒	A description of all covariates tested
☐	☒	A description of any assumptions or corrections, such as tests of normality and adjustment for multiple comparisons
☐	☒	A full description of the statistical parameters including central tendency (e.g. means) or other basic estimates (e.g. regression coefficient) AND variation (e.g. standard deviation) or associated estimates of uncertainty (e.g. confidence intervals)
☐	☒	For null hypothesis testing, the test statistic (e.g. F, t, r) with confidence intervals, effect sizes, degrees of freedom and P value noted<br>*Give P values as exact values whenever suitable.*
☒	☐	For Bayesian analysis, information on the choice of priors and Markov chain Monte Carlo settings
☒	☐	For hierarchical and complex designs, identification of the appropriate level for tests and full reporting of outcomes
☒	☐	Estimates of effect sizes (e.g. Cohen's d, Pearson's r), indicating how they were calculated

Our web collection on statistics for biologists contains articles on many of the points above.

Software and code

Policy information about availability of computer code

Data collection	Data from the dimethyl sulfide (DMS) analyzer were collected by using a data logger (USB-1408FS, Measurement Computing), and the collected csv data were processed by using Excel 2016 (Microsoft).
Data analysis	Data analysis was conducted using Origin 2019 or Origin 2022 (OriginLab Co.).

For manuscripts utilizing custom algorithms or software that are central to the research but not yet described in published literature, software must be made available to editors and reviewers. We strongly encourage code deposition in a community repository (e.g. GitHub). See the Nature Portfolio guidelines for submitting code & software for further information.

Data

Policy information about availability of data

All manuscripts must include a data availability statement. This statement should provide the following information, where applicable:

- Accession codes, unique identifiers, or web links for publicly available datasets
- A description of any restrictions on data availability
- For clinical datasets or third party data, please ensure that the statement adheres to our policy

The data that support the findings of this study are available from the corresponding author upon reasonable request.

March 2021

Human research participants

Policy information about studies involving human research participants and Sex and Gender in Research.

Reporting on sex and gender	This study did not involve human research participants.
Population characteristics	This study did not involve human research participants.
Recruitment	This study did not involve human research participants.
Ethics oversight	This study did not involve human research participants.

Note that full information on the approval of the study protocol must also be provided in the manuscript.

Field-specific reporting

Please select the one below that is the best fit for your research. If you are not sure, read the appropriate sections before making your selection.

☐ Life sciences ☐ Behavioural & social sciences ☒ Ecological, evolutionary & environmental sciences

For a reference copy of the document with all sections, see nature.com/documents/nr-reporting-summary-flat.pdf

Ecological, evolutionary & environmental sciences study design

All studies must disclose on these points even when the disclosure is negative.

Study description	The aim of this study was to determine whether Lake Baikal phytoplankton produce dimethylsulfoniopropionate (DMSP). Samples of the surface water of ice holes and plankton-colony water were taken for measurement of DMSP concentrations. The necessary equipment and measurement instruments were set up in March 2019, and measurements commenced on March 31. The last measurement on the ice was obtained on April 30, and shore water analysis continued until May 13. Fixed-point measurements were repeated almost every day, and surface-water samples were obtained on 24 days from point A (offshore) and on 29 days from point B (nearshore). Measurements at Point A ceased on April 24 because the site was no longer accessible. DMSP measurements were conducted twice for each sample. DMSP concentration changes from the start to the end of a plankton bloom were obtained. The correlation of DMSP concentration with plankton density was analyzed on the basis of 55 measurement data. For comparison, a summer campaign was carried out to measure DMSP concentration and plankton counts in 15 surface-water samples taken from a boat.
Research sample	The research samples were surface water taken from ice holes. The dominant plankton species was the dinoflagellate Gymnodinium baicalense, which was present at abundances of 20–4790 cell/mL in the surface water of the holes. Plankton colony waters were also collected by inserting a pipet into columnar cavities formed in ice. Suspended plankton could be present in both types of water samples.
Sampling strategy	The main purpose of this study was to investigate whether plankton in fresh water produce DMSP during the ice season. The first stage of the work was to determine whether DMSP could be detected in the lake water. As the second stage, instead of sample-size calculation, a series of DMSP concentration trends was obtained from the beginning to the end of the bloom. Monitoring was performed at two locations simultaneously to provide better data reliability. The same trends were observed at both locations, and the sampling points were sufficiently far apart to avoid bloom interaction.<br>The correlation between DMSP concentration and plankton density was obtained for sample waters taken from the two fixed sampling points, other holes, and clacks. The measurements were performed for more than 50 samples to obtain the correlation in the blooming season.
Data collection	Dr. Obolkin prepared ice holes for fixed-point monitoring in the middle of March 2019. Dr. Toda visited Baikal Museum (the building was previously the Limnological Institute before that institute moved to Irkutsk, and Dr. Obolkin still has his office there) in March and set up the DMS measurement device that had been developed as part of previous work (Anal. Chem. 2006, 78, 6252). The calibration curve was linear (R2 = 0.9988 in the range of 0-100 nM) and calibration range could be enlarged by changing the voltage applied to photomultiplier tube if necessary. The sensitivity was checked every morning with 50 nM DMSP standard solution and day-to-day variation was 4.8% during the campaign. The instrument raw signal was recorded by a data logger. Dr. Ohira joined the research at the beginning of April. The three researchers went out for water sampling and ice observation together. Two Japanese students, who were called Mr. Tashima (who traveled with Dr. Toda) and Mr. Abe (who traveled with Dr. Ohira), helped with DMSP analysis. Dr. Ohira counted plankton in a different room. After Dr. Toda had left, Dr. Ohira continued DMSP measurement with Dr. Obolkin. Dr. Saeki measured ionic components and amino acids. Dr. Ohira did the summer measurements with his student (Mr. Abe) and Dr. Obolkin.
Timing and spatial scale	Day-to-day variations of DMSP concentration were investigated from the end of March to the end of April, 2019. Sampling and measurement were performed every day, except for two days on which the weather was too bad for us to be out on the ice. Before the 2019 campaign, we carried out preliminary studies five times in 2011–2018 (in shorter periods of 5–10 days each, in March or April) and confirmed that the bloom maximum was around April 10 and DMSP concentration was high on those days and very low in

	March. Therefore, we decided to monitor DMSP from the end of March and to continue until accessing the sampling point was no longer possible.
Data exclusions	No data were excluded.
Reproducibility	The study was continued for a month to obtain day-to-day variations of DMSP concentration. We performed several preliminary surveys from 2011 onward, from which we came to know that DMSP levels are low in March and high in mid-April. Thus, the measurement period was set as the end of March until the ice melted, and we obtained the variations as expected. To confirm the tendency, two sampling points were used; the same trends were obtained from the two independent points. DMSP and plankton were measured twice and thrice, respectively, and the obtained values did not exhibit unreasonable differences.
Randomization	Sampling and measurements were performed from the beginning to the end of blooming; thus, the entire blooming period was investigated. Sampling was performed at a fixed time, 10–11 am, to minimize diurnal variation effects.
Blinding	Although DMSP and plankton measurements were carried out in the same institute, the people performing the measurements avoided communication about the current values being collected. DMSP was measured in Dr. Obolkin's laboratory and plankton were counted in a different laboratory that contained the microscope used. The measurement data were provided solely to the organizer of the study, Dr. Toda.

Did the study involve field work? ☒ Yes ☐ No

Field work, collection and transport

Field conditions	The main sampling points were holes prepared on the ice. The initial ice thickness (at the end of March) was ca. 70 cm. Details of meteorological data are included in Figure 2 of the paper. The ambient temperature was below freezing point every morning until April 20, after which daily minimum temperatures were mostly above freezing point.
Location	Two ice holes were prepared as fixed sampling points. Offshore point A was 700 m from shore at N 51°51′29.8″, E 104°50′04.2″, and nearshore point B was 70 m from the shore at N 51°51′52.1″, E 104°50′27.8″. Water depths were 650 m at point A and 10 m at point B.
Access & import/export	The sampling points were located approximately a 15-minute walk on ice from the base, Baikal Museum. Dr. Obolkin, whose research office is located in the museum, was responsible for the field activity. The Limnological Institute and Kumamoto University exchanged an agreement regarding the collaboration "Research of biogenic compounds produced by phytoplankton of Lake Baikal during subglacial blooms". The signing date was April 2, 2018, and the duration of the agreement was until December 31, 2020. No biological samples were imported or exported.
Disturbance	Two ice holes were made for fixed-point monitoring. People or animals do not walk on that area of the ice; however, tree branches were inserted into the ice as a warning signal to prevent accidents. Cars were not used for transport on the ice, to prevent disturbance.

Reporting for specific materials, systems and methods

We require information from authors about some types of materials, experimental systems and methods used in many studies. Here, indicate whether each material, system or method listed is relevant to your study. If you are not sure if a list item applies to your research, read the appropriate section before selecting a response.

Materials & experimental systems

n/a	Involved in the study
☒	☐ Antibodies
☒	☐ Eukaryotic cell lines
☒	☐ Palaeontology and archaeology
☒	☐ Animals and other organisms
☒	☐ Clinical data
☒	☐ Dual use research of concern

Methods

n/a	Involved in the study
☒	☐ ChIP-seq
☒	☐ Flow cytometry
☒	☐ MRI-based neuroimaging
