# Supplementary material for: Abundant production of dimethylsulfoniopropionate as a cryoprotectant by freshwater phytoplanktonic dinoflagellates in ice-covered Lake Baikal
Source: Commun Biol. 2023 Nov 24;6:1194. doi: 10.1038/s42003-023-05573-9 (PMC10674015; doi:10.1038/s42003-023-05573-9)
Supplement: Supplementary file 3 — Description of Supplementary Materials [file 42003_2023_5573_MOESM3_ESM.pdf]

Description of Additional Supplementary Files

File name: Supplementary Data

Description: The source data of graphs for Fig. 2abc and Fig. 3ab.
